# The potential protective effect of *Commelina nudiflora* L. against carbon tetrachloride (CCl_4_)-induced hepatotoxicity in rats, mediated by suppression of oxidative stress and inflammation

**DOI:** 10.1186/s12199-017-0673-0

**Published:** 2017-09-11

**Authors:** Muhammad Dawood Shah, Urban J.A. D’Souza, Mohammad Iqbal

**Affiliations:** 1 Biotechnology Research Institute, Universiti Malaysia Sabah, Jalan UMS, 88400 Kota Kinabalu, Sabah Malaysia; 2 Faculty of Medicine and Health Science, Universiti Malaysia Sabah, Jalan UMS, 88400 Kota-Kinabalu, Sabah Malaysia

**Keywords:** *C. nudiflora*, Hepatoprotection, Antioxidant enzymes, Acute liver injury, Bioactive compounds, Immunohistochemistry

## Abstract

**Background:**

This study aims to assess the hepatoprotective potential of *Commelina nudiflora* against CCl_4_-induced hepatic injury in rats.

**Method:**

Antioxidant activities were determined. Phytochemical analysis was performed by gas chromatography mass spectrometry (GCMS). In the in vivo study, Sprague Dawley rats were pretreated with *C. nudiflora* (150, 300, and 450 mg kg body weight (b.wt.)) once daily for 14 days followed by two doses of CCl_4_ (1 ml/kg b.wt.). After 2 weeks, the rats were sacrificed and hepatoprotective analysis was performed.

**Results:**

In vitro studies have shown that the extract possessed strong antioxidant activity and has ability to scavenge 2,2-diphenyl-2-picrylhydrazyl-free radicals effectively. GCMS analysis of the *C. nudiflora* extract revealed the presence of various bioactive compounds. Administration of *C. nudiflora* significantly reduced the impact of CCl_4_ toxicity on serum markers of liver damage, serum aspartate transaminase (AST), and alanine transaminase (ALT). *C. nudiflora* also increased antioxidant levels of hepatic glutathione (GSH) and antioxidant enzymes and ameliorated the elevated hepatic formation of malondialdehyde (MDA) induced by CCl_4_ in rats. Histopathological examination indicated that *C. nudiflora* protect the liver from the toxic effect of CCl_4_ and healed lesions such as necrosis, fatty degeneration, and hepatocyte injury as irregular lamellar organization and dilations in the endoplasmic reticulum. The immunohistochemical studies revealed that pretreatment of *C. nudiflora* decreased the formation of 4-hydroxy-2-nonenal (HNE)-modified protein adducts and 8-hydroxy-2′-deoxyguanosine (8-OHdG). Furthermore, overexpression of the proinflammatory cytokines TNF-α, IL-6, and prostaglandin E2 is also reduced.

**Conclusion:**

These findings exhibited the potential prospect of *C. nudiflora* as functional ingredients to prevent ROS-related liver damage.

## Background

The liver plays a vital role in the metabolism, detoxification, and removal of different toxic chemicals from the body. The regulation of different physiochemical functions, such as oxidation, reduction, hydroxylation, hydrolysis, conjugation, sulfation, and acetylation, happens in the liver. Currently, liver diseases pose serious health issues. Environmental pollutants, infectious agents, and hepatotoxins (carbon tetrachloride) are reported to cause liver injuries [1, 2]. Carbon tetrachloride (CCl_4_) is a compound that is most commonly used to induce liver injuries in experimental animals [3]. Synthetically prepared or conventional drugs used for the prevention and treatment of liver injuries might be inadequate and can cause serious side effects. Due to these reasons, a large number of people around the world prefer to consume herbal plants for the treatment of liver diseases. Thus, it is important to find alternative drugs from natural plants with less toxicity and high efficacy for the treatment of liver disorders [4].

*Commelina nudiflora*, which belongs to the family Commelinaceae, is a slender and perennial herbal plant. It is native to Malaysia, India, Bangladesh, and other tropical Asian countries. The stem of the plant is 15–30 cm long, with green leaves and purple flowers. It is mostly found in wet places [5]. The plant is used in the treatment of intestinal obstruction, diarrhea, hemorrhoids, abnormal uterine bleeding, and vaginal discharge. In addition to that, it is also used to cure wart and erysipelas (deep red inflammation of skin). In East Africa, *C. nudiflora* is consumed for sore throats, while in India, the plant is believed to be beneficial in the treatment of leprosy [5, 6]. However, there have been no scientific studies on the chemical composition and hepatoprotective effects of *C. nudiflora*. Therefore, this study aims to evaluate the phytochemical constituents and chemopreventive effects of *C. nudiflora* against CCl_4_-induced oxidative stress and hepatic dysfunction in rats to determine possible hepatoprotective activity.

## Methods

### Chemicals, antibodies, and kit

CC1_4_, Tris-HCl, thiobarbituric acid (TBA), oxidized and reduced glutathione, reduced β-nicotinamide adenine dinucleotide phosphate (NADPH), glucose-6-phosphate, 1-chloro-2,4-dinitrobenzene (CDNB), glutathione reductase, 5,5-dithio-bis-2-nitrobenzoic acid (DTNB), 2,2′-azinobis-(3-ethylbenzothiazoline-6-sulfonic acid (ABTS), potassium persulfate, sulfosalicylic acid (SSA), bovine serum albumin (BSA), hydrogen peroxide (H_2_O_2_), flavin adenine dinucleotide (FAD), 2,6-dichloroindophenol, trichloroacetic acid (TCA), Tween 20, and ethylenediaminetetraacetic acid (EDTA) were obtained from Sigma Chemical Co. (St. Louis, MO, USA). Rabbit polyclonal antibody specific for 8-hydroxy-2′-deoxyguanosine (8-8-OHdG), rabbit polyclonal antibody specific for 4-hydroxy-2-nonenal (HNE), rabbit polyclonal antibody specific to tumor necrosis factor alpha (TNF-α), rabbit polyclonal antibody specific to interleukin 6 (IL-6), rabbit polyclonal antibody specific to prostaglandin E2 (PGE2), EnVision™ + System/horseradish peroxidase (HRP), Rb (DAB+), target retrieval solution, and antibody diluent were purchased from Dako (Agilent Technologies Company, Denmark).

### Plant collection and extraction

The plant was collected from the lowlands of Papar, Sabah, Malaysia. Plant identification was confirmed by Mr. Kulip and Mr. Johnny Gisil from the Institute of Tropical Biology and Conservation, Universiti Malaysia Sabah. A voucher specimen (MDS003) was deposited at the Tropical Biology and Conservation Herbarium, Universiti Malaysia Sabah. Sixty grams of dry powder was extracted with 300 ml of methanol by the Soxhlet method (50–60 °C and 72 h). Methanol residues were removed from the extract using a vacuum rotary evaporator. The samples were kept at − 80 °C for 24 h and then lyophilized using a freeze dryer. The freeze-dried samples were then stored in the freezer for further analysis [7].

### Total phenolic content

The total phenolic content in the *C. nudiflora* methanol extract was determined by the Folin-Ciocalteu method [8] with slight modifications. A stock solution of 1 mg/ml was prepared from the extract. A Folin-Ciocalteu reagent was prepared by tenfold dilution (ratio 1:9). The reagent reacts with phenolic and non-phenolic reducing substances to form chromogens that can be measured spectrophotometrically [8]. Briefly, 1.5 ml of Folin-Ciocalteu reagent was mixed with 0.2 ml of assay samples and mixed vigorously. After 5 min, 1.5 ml of sodium carbonate (60 g/l) was added to the mixture. Finally, the mixture was allowed to stand for 90 min in the dark at room temperature. The absorbance was measured at 725 nm against a blank. Gallic acid was used as a standard for the quantification of phenolic compounds. Concentrations of 0.01, 0.02, 0.04, 0.08, and 0.1 mg/ml of gallic acid were used to plot the standard calibration curve. The concentration of the total phenolic content was estimated as milligrams of gallic acid equivalent by using an equation obtained from the gallic acid calibration curve.

### 2,2-Diphenyl-2-picrylhydrazyl assay

The antioxidant activity of *C. nudiflora* methanol extracts was determined via 2,2-diphenyl-2-picrylhydrazyl (DPPH) assay [9]. A plant extract was prepared at a concentration of 1 mg/ml. Various concentrations (0.012, 0.025, 0.050, 0.1, and 0.5 mg/ml) of plant extract were used. During the process, 0.3 ml of plant extract was mixed with 2.7 ml of DPPH (6 × 10^−5^ M) in methanol and left in the dark for 60 min. Absolute methanol was used as a blank. The absorbance was measured at 512 nm using a spectrophotometer. Ascorbic acid was used as a standard. The radical scavenging activity was calculated according to the formula summarized below

$$ \%\mathrm{RSA}=\left[\left({A}_{\mathrm{B}\ \mathrm{control}}-{A}_{\mathrm{A}\ \mathrm{sample}}\right)/{A}_{\mathrm{B}\ \mathrm{control}}\right]\times 100 $$

where % RSA is the percentage of radical scavenging activities, *A*_A_ is the absorbance values of the extract sample, and *A*_B_ is the absorbance values of the control sample.

### Gas chromatography mass spectrometry analysis of *C. nudiflora*

A small quantity of *C. nudiflora* methanol extract was injected into a gas chromatography mass spectrometry (GCMS) system, which consisted of an Agilent 7890A gas chromatograph system coupled with an Agilent 5975C mass spectrometry detector. A capillary column, HP-5MS (30 m × 0.25 mm) of 0.25 μm film thickness of coated material, was used. The injector temperature was set at 250 °C whereas the temperature settings were as follows: start at 40 °C and hold for 3 min; from 40 to 200 °C with 3 °C/min and then hold for 3 min. A post-run of 5 min at 200 °C was sufficient for the next injection. A gas chromatography was performed in splitless mode. Helium gas was used as a carrier gas and maintained at a 1.0 ml/min constant flow rate. Identification of various compounds was carried out by referring to the NIST library, and the chemical makeup was computed with reference to the abundance of the compounds in a chromatogram. Each analysis was carried out in triplicate, together with a blank solvent.

### Experimental protocol

Male Sprague Dawley rats weighing 150–250 g were utilized throughout the experiment. The animals were acquired from the Animal Breeding House, Biotechnology Research Institute, Universiti Malaysia Sabah. The animals were maintained at a room temperature (25 °C) in a temperature-controlled room and allowed access to food (normal laboratory chow) and tap water ad libitum. All the animals were treated humanely and well maintained under standard ethical principles as per university regulations (UMS/IP7.5/M3/4-2012). They were acclimatized to laboratory conditions for 7 days before experiments started. CCl_4_ was prepared at a dose of 1.0 ml/kg body weight with corn oil (1:1). A suspension of plant extract was prepared in distilled water, and different doses of *C. nudiflora* extract (150, 300, and 450 mg/kg body weight) were administered to the animals by gastric gavage needles. Thirty-six adult male rats were taken and distributed randomly into six groups of six animals each. Group 1 served as a normal control; group 2 was treated with CCl_4_ (1.0 ml/kg body weight); groups 3, 4, and 5 were treated with *C. nudiflora* (150, 300, and 450 mg/kg body weight, respectively) + CCl_4_ (1.0 ml/kg body weight); and group 6 was treated only with high doses of *C. nudiflora* (450 mg/kg body weight).

The rats were pretreated with selected doses of *C. nudiflora* methanol extracts continuously for 14 days, followed by an administration of CCl_4_ on the 13th and 14th days. The doses of the plant extract (150, 300, and 450 mg/kg body weight) and CCl_4_ (1.0 ml/kg body weight) were administered. All of these rats were sacrificed 24 h after the last dose of CCl_4_ within a period of 1 h. Blood was collected by cardiac puncture using sterile disposable syringes, while serum was obtained by centrifugation at 2000×*g* for 15 min. The livers of these animals were removed immediately and cleaned with chilled saline (0.85% *w*/*v*, sodium chloride) to remove an extrinsic material. The liver tissues were stored at − 80 °C for biochemical studies while a small portion of the tissues was kept in a 10% neutral buffered formalin solution for histopathological and immunohistochemical analyses.

### Preparation of post-mitochondrial and cytosolic supernatant

The hepatic homogenate was prepared by the method proposed by Mohandas et al. [10], as described by Iqbal et al. [11]. The rat livers were homogenized in an ice-cold phosphate buffer (0.1 M, pH 7.4) containing KCl (1.17% *w*/*v*) using a homogenizer (Polytron PT 1200E, Switzerland). The nuclear debris was removed from the liver homogenate by centrifugation at 3000×*g* for 10 min at 4 °C. The post-mitochondrial supernatant (PMS) was obtained by centrifugation at 12,000×*g* for 30 min at 4 °C, which was utilized for the measurement of malondialdehyde (MDA) and reduced glutathione (GSH) content, as well as a source of antioxidant enzymes. A part of PMS was further ultracentrifuged at 105,000×*g* for 1 h to obtain cytosolic fractions to determine quinone oxidoreductase assay activity.

### Biochemical assays

#### Assay of reduced glutathione

Reduced glutathione in liver PMS was estimated according to the method described by Jollow et al. [12]. Briefly, 1.0 ml of hepatic PMS (10% *w*/*v*) was reacted with 1.0 ml of sulfosalicylic acid (4% *w*/*v*). The samples were kept at 4 °C for 60 min and then centrifuged at 3000×*g* for 30 min at 4 °C. The assay mixture contained 0.2 ml filtered supernatant, 2.6 ml phosphate buffer (0.1 M, pH 7.4), and 0.2 ml DTNB (4 mg/ml of 0.1 M phosphate buffer, pH 7.4) in a total volume of 3.0 ml. The development of a yellow color was read immediately at 412 nm on a spectrophotometer (model 4001/4). Results were expressed as micromoles of reduced glutathione per gram of liver tissue.

#### Assay of lipid peroxidation

Hepatic lipid peroxidation in PMS was performed by the method described by Buege and Aust [13]. Briefly, 1.0 ml of PMS was mixed with 0.5 ml of trichloroacetic acid (10% *w*/*v*) and centrifuged at 800×*g* for 30 min, and 1.0 ml of the supernatant was reacted with 1.0 ml of thiobarbituric acid (0.67% *w*/*v*). All the tubes were placed in a boiling water bath for a time period of 20 min. The tubes were then transferred to an ice bath and allowed to cool for 5 min. The amount of MDA formed in each of the samples was assessed by measuring the optical density of the supernatant at 535 nm using a spectrophotometer (model 4001/4). The results were expressed as nanomoles of MDA formed/gram tissue using a molar extinction coefficient of 1.56 × 10^5^ M × 1 cm × 1.

#### Assay of glutathione peroxidase activity

Glutathione peroxidase activity in liver PMS was carried out according to the method by Mohandas et al. [10] as described by Iqbal et al. [14]. Briefly, 0.025 ml of 10% *w*/*v* hepatic PMS was added to a reaction mixture that consisted of 1.51 ml of 0.1 M phosphate buffer (pH 7.4), 0.1 ml EDTA (0.5 mM), 0.1 ml sodium azide (1.0 mM), 0.05 ml glutathione reductase (1.0 EU/ml), 0.1 ml GSH (1.0 mM), 0.1 ml NADPH (0.1 mM), and 0.01 ml hydrogen peroxide (30%), forming a total volume of 2.0 ml. Enzyme activity was calculated as nanomoles of NADPH oxidized/minute/milligram protein using a molar extinction coefficient of 6.22 × 10^3^ M × 1 cm × 1.

#### Assay of glucose-6-phosphate dehydrogenase activity

Glucose-6-phosphate dehydrogenase activity was measured by the method of Zaheer et al. [15] as described by Iqbal et al. [14]. Briefly, a reaction mixture of 3.0 ml, consisting of 0.5 ml of 0.05 M Tris-HCl buffer (pH 7.6), 0.05 ml NADP (0.1 mM), 0.05 ml glucose-6-phosphate (0.8 mM), 0.25 ml MgCl_2_ (8 mM), 0.1 ml of 10% *w*/*v* hepatic PMS, and 2.0 ml of distilled water, was prepared. The changes in absorbance were noted at 340 nm, and the enzyme activity was calculated as nanomoles of NADP reduced/minute/milligram protein using a molar extinction coefficient of 6.22 × 10^3^ M × 1 cm × 1.

#### Assay of glutathione reductase activity

Glutathione reductase activity was determined by the Carlberg and Mannervik method, [16] as described by Iqbal et al. [14]. Briefly, 0.05 ml of 10% *w*/*v* hepatic PMS was mixed with 1.7 ml of phosphate buffer (0.1 M, pH 7.6), 0.1 ml EDTA (0.5 mM), 0.05 ml oxidized glutathione (1 mM), and 0.1 ml NADPH (0.1 mM) and the reaction mixture was read at 340 nm. Enzyme activity was calculated as nanomoles of NADPH oxidized/minute/milligram protein using a molar extinction coefficient of 6.22 × 10^3^ M × 1 cm × 1.

#### Assay of catalase activity

Catalase activity was determined by the Claiborne [17] method, as described by Iqbal et al. [14]. Briefly, a reaction mixture of 2 ml, consisting of 0.99 ml of 0.05 M phosphate buffer (pH 7.0), 1.0 ml of 0.019 M hydrogen peroxide, and 0.01 ml of hepatic PMS (10% *w*/*v*), was prepared. The changes in absorbance of the reaction solution were noted at 240 nm, and the enzyme activity was calculated as nanomoles of H_2_O_2_ consumed/minute/milligram protein using a molar extinction coefficient of 6.4 × 10^3^ M × 1 cm × 1.

#### Assay of glutathione *S*-transferase activity

Glutathione *S*-transferase activity was measured by the Habig et al. [18] method, as modified by Athar and Iqbal [19]. The assay system was obtained by the addition of 2.75 ml phosphate buffer (0.1 M, pH 6.5), 0.1 ml reduced glutathione (1.0 mM), 0.1 ml CDNB (1.0 mM), and 0.25 ml of hepatic PMS (10% *w*/*v*). The absorbance was determined at 340 nm, and the enzyme activity was calculated as nanomoles of CDNB conjugate formed/minute/milligram protein using a molar extinction coefficient of 9.6 × 10^3^ M × 1 cm × 1.

#### Assay of NAD(P)H: quinoneoxido reductase activity

Quinone reductase activity was determined by the Benson et al. [20] method, as modified by Iqbal et al. [21]. The assay mixture consisted of 2.0 ml of Tris-HCl buffer (0.025 M, pH 7.4), 0.7 ml BSA (1 mg/ml), 0.1 ml FAD (150 μM), 0.02 ml NADPH (0.1 mM), 0.02 ml Tween 20 (1% *w*/*v*), 0.05 ml of cytosolic fraction (10% *w*/*v*), and 0.05 ml of 2,6-dichlorophenolindophenol (2.4 mM) in a final volume of 3.0 ml. The enzyme activity was determined at 600 nm and calculated as nanomoles of 2,6-dichlorophenolindophenol reduced/minute/milligram protein using a molar extinction coefficient of 2.1 × 10^4^ M × 1 cm × 1.

#### Assay of serum alanine transaminase and aspartate transaminase

Serum alanine transaminase (ALT) and aspartate transaminase (AST) levels were determined by the method described by Reitman and Frankel [22]. Briefly, 0.5 ml of α-ketoglutarate (2 mM) and α-l-alanine (200 mM) for ALT, and α-ketoglutarate (2 mM) and l-aspartate (200 mM) for AST, was incubated in a water bath for 10 min at 37 °C; 0.1 ml of serum was added, and the volume was made up to 1.0 ml with a sodium phosphate buffer. The reaction mixture was incubated for exactly 30 and 60 min for ALT and AST, respectively. After that, 0.5 ml of DNPH (1 mM) was added to the reaction mixture and kept for another 20 min at room temperature. Finally, the change in the color was noted by the addition of 5.0 ml of NaOH (0.4 N) and the final product was read at 510 nm after 30 min.

#### DPPH assay

The free radical scavenging activity of rat tissue and serum samples was determined by the DPPH method, described by Brand-Williams et al. [23] with slight modifications. Briefly, 100 μl of each sample was added to 2.7 ml of DPPH in an ethanol solution (6 × 10^−5^ M) in a test tube. The tubes were kept in the dark for 1 h. After that, the samples were treated with 1 ml of chloroform and centrifuged at 1107×*g* for 5 min. The absorbance of clear solution was recorded at 517 nm using a spectrophotometer. An ethanol solution of DPPH (6 × 10^−5^ M) without the sample was used as a control, and the percentage of DPPH radical scavenging activity was calculated according to the following equation:

$$ \%\mathrm{DPPH}=\left[\left({A}_{\mathrm{B}\ \mathrm{control}}-{A}_{\mathrm{A}\ \mathrm{sample}}\right)/{A}_{\mathrm{B}\ \mathrm{control}}\right]\times 100 $$

where *A*_A_ is the absorbance values of the extract sample and *A*_B_ is the absorbance values of the control sample.

### ABTS assay

The free radical scavenging activity of our samples was performed via ABTS assay, as proposed by Re et al. [24]. Briefly, a stock solution of ABTS (7 mM) was prepared in water. An ABTS radical cation (ABTS_S+_) was obtained by treating ABTS stock solution with 2.45 mM potassium persulfate (final concentration). The mixture was kept in the dark overnight (12–16 h). The ABTS_S+_ solution was then diluted with phosphate buffer saline with a pH value of 7.4 (PBS) to an absorbance of 0.70 at 734 nm. After that, 20 μl of tissue or serum was added to 2 ml of diluted ABTS_S+_ solution. The mixture was incubated for 6 min at 30 °C, and the absorbance was recorded at 734 nm. ABTS solution without the sample was used as a control. The percentage inhibition of ABTS_S+_ by the sample was calculated according to the given equation:

$$ \%\mathrm{ABTS}=\left[\left({A}_{\mathrm{B}\ \mathrm{control}}-{A}_{\mathrm{A}\ \mathrm{sample}}\right)/{A}_{\mathrm{B}\ \mathrm{control}}\right]\times 100 $$

where *A*_A_ is the absorbance values of the extract sample and *A*_B_ is the absorbance values of the control sample.

### Histopathological studies in liver tissue

#### Light microscopy analysis

For histopathological studies, a fixed portion of rat liver in 10% neutral buffered formalin solution was dehydrated in alcohol and embedded in paraffin. Cut thin sections (5–6 μm) were placed on glass slides and stained with hematoxylin and eosin (H&E) stains. The slides were examined under a light microscope by an expert pathologist who was not aware of the sample assignments to experimental groups for the pathological symptoms of hepatotoxicity, such as necrosis, hepatocyte derangement, fatty degeneration, and blood vessel congestion.

#### Transmission electron microscopy analysis

Small sections of hepatic tissues were fixed in 4% glutaraldehyde in 0.1 M of phosphate buffer for electron microscopy evaluations. The samples were washed and post-fixed in 1% osmium tetroxide (0.1 M phosphate buffer). Afterwards, the tissues were dehydrated in increasing concentrations of alcohol. Finally, the tissues were washed with 0.1 M of phosphate buffer and embedded in epoxy-resin embedding media. Ultrathin sections were obtained on copper grids, stained with uranyl acetate and lead citrate, and viewed under a transmission electron microscope.

### Immunohistochemistry

Immunohistochemical studies were conducted using a Dako Kit. During the process, five antigens, which were HNE-modified protein adducts, 8-OHdG, TNF-α, IL-6, and PGE2, were screened. The hepatic tissues were fixed in 10% phosphate-buffered formalin for 24 h and embedded in paraffin. The paraffin sections were then de-paraffinized and rehydrated through a xylene and graded ethanol series. Antigen retrieval was performed using a water bath at 97 °C for 20 min. The samples were further treated with 3.0% hydrogen peroxide for 5 min and rinsed with Tris-buffered saline (TBS). The tissue sections on the slide were highlighted by wax pens in order to define the spot for the application of various staining reagents. The samples were then incubated for 20 min at room temperature with diluted antibodies, rinsed with TBS three times for 10 min, and applied with HRP for 20 min. The slides were rinsed again with TBS three times for 10 min, and freshly prepared DAB working solution was applied for 5–10 min, followed with a rinse by deionized water three times. The slides were counterstained by Harris hematoxylin for one dip and dehydrated through a graded ethanol and xylene series. The staining intensities of these markers were evaluated semi-quantitatively (weak/strong) and compared among the six groups.

### Assay of protein

Protein concentration in all samples was determined by a bicinchoninic acid (BCA) protein assay kit using BSA as a standard.

### Statistical analysis

The data was analyzed using SPSS 17.0 Windows statistical package software (SPSS, Inc., Chicago, IL, USA). Significant differences between groups were analyzed using one-way analysis of variance (ANOVA) followed by Tukey’s multiple comparison test. All the results were presented as means ± standard error (S.E.) of the mean. *p* values less than 0.05 were considered as significant.

## Results

### Total phenolic and DPPH scavenging activity of *C. nudiflora*

**Fig. 1:**
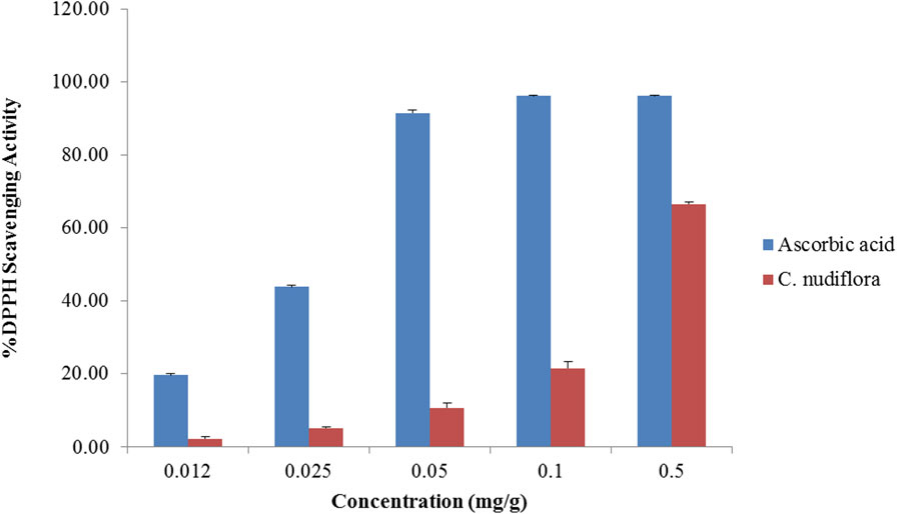
Free radical scavenging capacity of the methanol extract of *C. nudiflora*. Each value represents the mean ± S.E. of triplicate tubes. Experimental conditions are described in the “Methods” section

The methanol extract of *C. nudiflora* was found to contain 42.67 ± 1.78 mg/g total phenolics expressed as gallic acid equivalents (GAEs, mg/g of extract). DPPH has been widely utilized to determine the scavenging activity of bioactive compounds. The DPPH scavenging capacity of the methanol extract of *C. nudiflora* increased in a concentration-dependent manner. The concentration of the plant ranged from 0.012 to 0.5 mg/ml, and the percentage of DPPH scavenging capacity ranged from 2.32 to 66.44% (Fig. 1).

### GCMS analysis of *C. nudiflora*

**Fig. 2:**
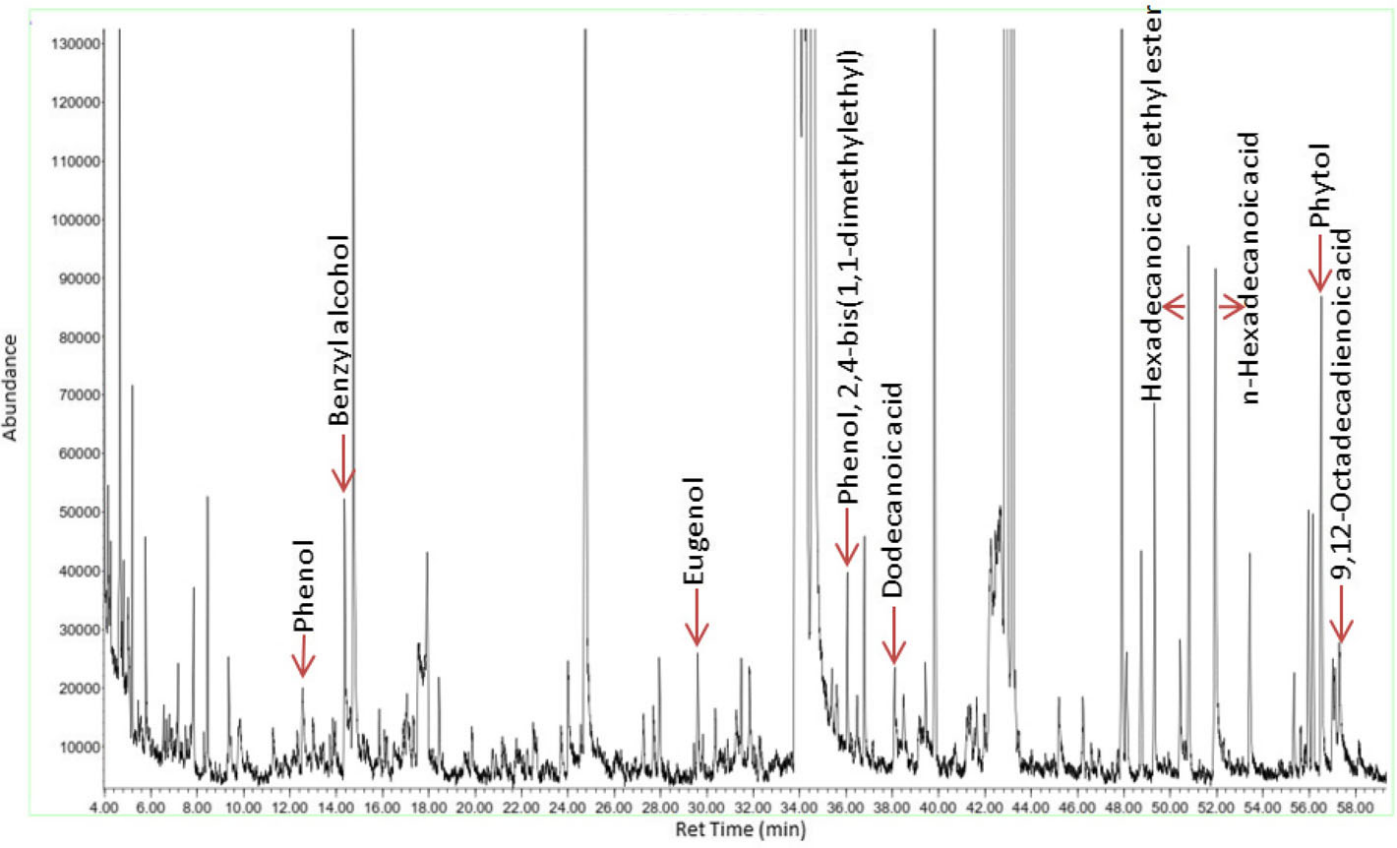
GCMS chromatogram of the methanol extract of *C. nudiflora*

The GCMS analysis of phytochemical constituents was carried out in methanol extracts of *C. nudiflora*, as shown in Fig. 2. The bioactive compounds of various natures are tabulated along with retention time (ret time), molecular formula (M. formula), molecular weight (M. weight), and area percentage (%) (Table 1). The identified bioactive compounds include phenol (0.71%), benzyl alcohol (1.62%), eugenol (0.64%), phenol,2,4-bis(1,1-dimethylethyl) (0.77%), dodecanoic acid (0.70%), hexadecanoic acid, ethyl ester (2.27%), *n*-hexadecanoic acid (3.15%), phytol (2.61%), and 9,12-octadecadienoic acid (*Z*,*Z*)- (0.66%).

**Table 1:** Identified bioactive compounds in the methanol extract of *C. nudiflora*

No.	Ret. time	Name of the compounds	Nature	M. weight	M. formula	Area (%)
1	12.54	Phenol	Phenolic compound	94.11	C_6_H_6_O	0.71
2	14.35	Benzyl alcohol	Aromatic compound	108.13	C_7_H_8_O	1.62
3	29.06	Eugenol	Phenolic compound	164.20	C_10_H_12_O_2_	0.64
4	36.06	Phenol,2,4-bis(1,1-dimethylethyl)	Alkylated phenol	206.32	C_14_H_22_O	0.77
5	38.1	Dodecanoic acid	Fatty acid	200.31	C_12_H_24_O_2_	0.70
6	50.79	Hexadecanoic acid ethyl ester	Palmitic acid ester	284.0	C_18_H_36_O_2_	2.27
7	51.95	*n*-Hexadecanoic acid	Palmitic acid	256.42	C_16_H_30_O_2_	3.15
8	56.71	Phytol	Diterpene alcohol	296.53	C_20_H_40_O	2.61
9	57.10	9,12-Octadecadienoic acid (*Z*,*Z*)-	Linoleic acid	280.44	C_18_H_32_O_2_	0.66

### Effect of *C. nudiflora* on body weight

Table 2 shows the effect of *C. nudiflora* on rats’ body weight. CCl_4_ administration resulted in a reduction in body weight. However, the animals that were pretreated with *C. nudiflora* extracts (150, 300, and 450 mg/kg body weight) for 2 weeks followed by two doses of CCl_4_ on the 13th and 14th days recorded a moderate increase in body weight.

**Table 2:** Effect of *C. nudiflora* on body weight

Groups	Initial body weight (g)	Final body weight (g)	Percentage increase (%)
Saline control	224 ± 39.1	260.5 ± 33.5	16.2
CCl_4_ (1 ml/kg b.wt.)	224 ± 32.4	237 ± 32.6	5.8
*C. nudiflora* (150 mg/kg b.wt.) + CCl_4_	219.8 ± 45.9	237.5 ± 45.8	8.0
*C. nudiflora* (300 mg/kg b.wt.) + CCl_4_	223 ± 25.9	242.3 ± 15.3	8.6
*C. nudiflora* (450 mg/kg b.wt.) + CCl_4_	220.5 ± 45.3	240 ± 48.3	8.8
*C. nudiflora* (450 mg/kg b.wt.)	224 ± 28.2	259.5 ± 38.3	15.8

Each value represents the mean ± S.E. of six animals

### Effect of *C. nudiflora* on CCl_4_-induced hepatotoxicity

**Fig. 3:**
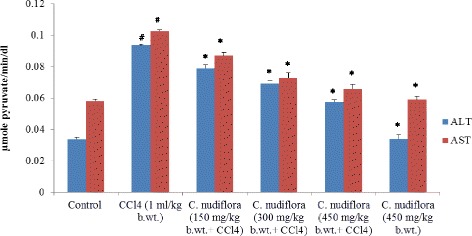
Effect of *C. nudiflora* on CCl_4_-induced hepatotoxicity. Each value represents the mean ± S.E. of six animals. Hashtag indicates significance at *p* < 0.05 compared to the control group. Asterisk indicates significance at *p* < 0.05 compared to the CCl_4_-treated group

The levels of ALT and AST (hepatic enzymes), used as markers for the determination of liver injuries, were significantly higher in CCl_4_-administered rats as compared with saline-treated control rats (Fig. 3). Meanwhile, the pretreatment of animals with *C. nudiflora* at doses of 150, 300, and 450 mg/kg body weight significantly decreased hepatic enzyme levels in a dose-dependent manner as compared with the CCl_4_-administered group.

### Effect of *C. nudiflora* on CCl_4_-induced hepatic lipid peroxidation

**Fig. 4:**
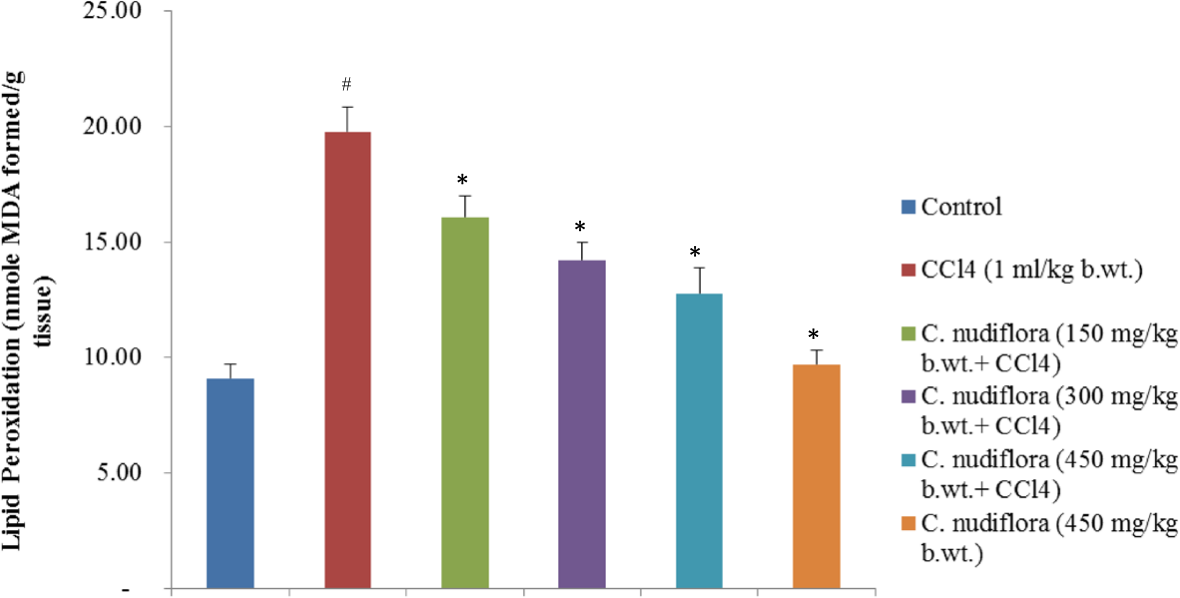
Effect of *C. nudiflora* on CCl_4_-induced hepatic lipid peroxidation. Each value represents the mean ± S.E. of six animals. Hashtag indicates significance at *p* < 0.05 compared to the control group. Asterisk indicates significance at *p* < 0.05 compared to the CCl_4_-treated group

MDA is the end product and a common marker of lipid peroxidation. Figure 4 shows that MDA levels are significantly elevated in the livers of CCl_4_-treated rats as compared to the saline-treated control rats. However, the pretreatment of methanol extracts of *C. nudiflora* significantly reduced MDA levels. These results suggest that oxidative stress induced by CCl_4_ was suppressed by the administration of methanolic extracts of *C. nudiflora*.

### Effect of *C. nudiflora* on CCl_4_-induced reduced glutathione

**Fig. 5:**
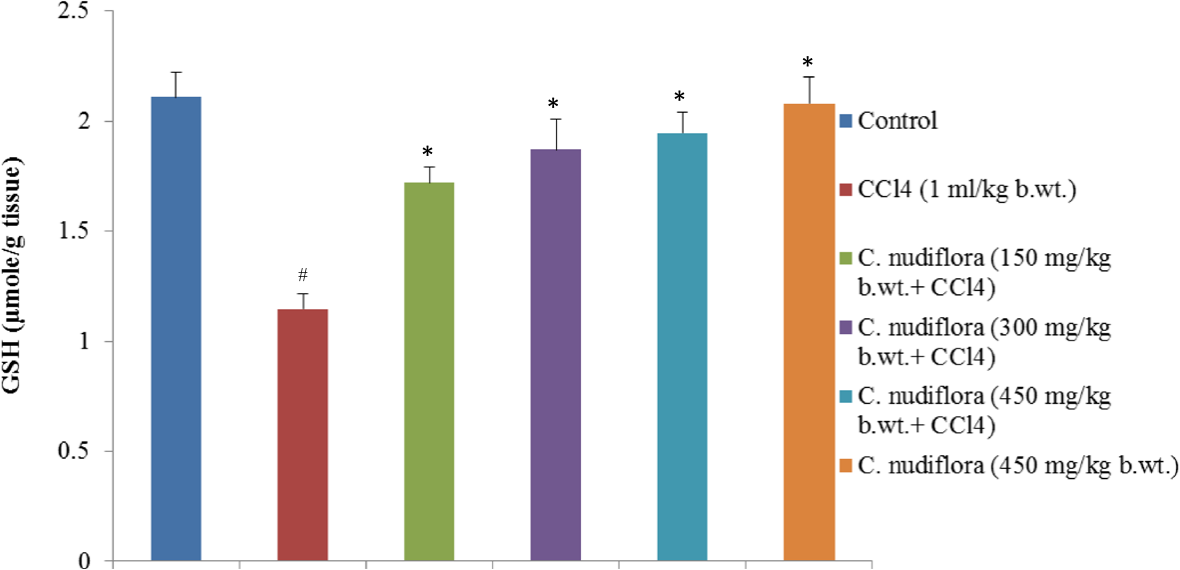
Effect of *C. nudiflora* on CCl_4_-induced hepatic reduced glutathione. Each value represents the mean ± S.E. of six animals. Hashtag indicates significance at *p* < 0.05 compared to the control group. Asterisk indicates significance at *p* < 0.05 compared to the CCl_4_-treated group

GSH levels in the tissue often decrease under a state of oxidative stress. Therefore, we studied the effects of *C. nudiflora* methanol extracts on liver GSH levels in the CCl_4_-administered group. Oxidative stress caused by CCl_4_ significantly reduced levels of hepatic GSH in that group. However, CCl_4_-administered animals treated with *C. nudiflora* (150, 300, and 450 mg/kg b.wt.) had significantly increased GSH levels by 27, 34, and 38%, respectively, as compared with CCl_4_-administered rats (Fig. 5).

### Effect of *C. nudiflora* on CCl_4_-induced changes in antioxidant enzymes

The activities of hepatic antioxidant enzymes, viz., catalase, glutathione peroxidase, glutathione reductase, and glucose-6-phosphate dehydrogenase, decreased in the CCl_4_-administered group as compared with the saline-treated control group (Table 3). The group treated with CCl_4_ also indicated reductions in the levels of phase II metabolizing enzymes (glutathione *S*-transferase, quinone reductase) (Table 4). Meanwhile, the pretreatment of *C. nudiflora* resulted in the restoration of antioxidant enzyme levels in a dose-dependent manner.

**Table 3:** Effect of *C. nudiflora* on CCl_4_-induced changes in antioxidant enzymes

Groups	Glutathione peroxidase (nmol NADPH oxidized/min/mg protein)	Glutathione reductase (nmol NADPH oxidized/min/mg protein)	Catalase (nmol H_2_O_2_ consumed/min/mg protein)	Glucose-6-phosphate dehydrogenase (nmol NADPH formed/min/mg protein)
Saline control	61.87 ± 1.93	50.61 ± 1.32	0.82 ± 0.01	40.05 ± 1.37
CCl_4_ (1 ml/kg b.wt.)	47.89 ± 2.06^#^	38.62 ± 1.05^#^	0.50 ± 0.12^#^	21.14 ± 1.67^#^
*C. nudiflora* (150 mg/kg b.wt.) + CCl_4_	55.18 ± 3.95*	45.64 ± 3.66*	0.68 ± 0.05*	33.35 ± 1.53*
*C. nudiflora* (300 mg/kg b.wt.) + CCl_4_	59.18 ± 2.08*	46.61 ± 1.03*	0.70 ± 0.04*	34.33 ± 1.41*
*C. nudiflora* (450 mg/kg b.wt.) + CCl_4_	61.17 ± 4.74*	47.56 ± 0.71*	0.74 ± 0.02*	35.80 ± 0.9*
*C. nudiflora* (450 mg/kg b.wt.)	61.99 ± 5.15*	50.11 ± 1.59*	0.81 ± 0.04*	40.15 ± 1.35*

Each value represents the mean ± S.E. of six animals

^#^Significant at *p* < 0.05 compared to the control group; *significant at *p* < 0.05 compared to the CCl_4_-treated group

**Table 4:** Effect of *C. nudiflora* on CCl_4_-induced changes in phase II metabolizing enzymes

Groups	Glutathione *S*-transferase activity (nmol CDNB conjugate formed/min/mg protein)	Quinone reductase activity (nmol 2,6-DCIP reduced/min/mg protein)
Saline control	253.55 ± 11.49	313.17 ± 27.7
CCl_4_ (1 ml/kg b.wt.)	118.44 ± 7.53^#^	161.99 ± 6.9^#^
*C. nudiflora* (150 mg/kg b.wt.) + CCl_4_	148.19 ± 2.63*	220.99 ± 15.4*
*C. nudiflora* (300 mg/kg b.wt.) + CCl_4_	174.76 ± 9.49*	240.62 ± 12.53*
*C. nudiflora* (450 mg/kg b.wt.) + CCl_4_	210.34 ± 16.06*	260.15 ± 6.14*
*C. nudiflora* (450 mg/kg b.wt.)	254.26 ± 9.90*	312.51 ± 11.97*

Each value represents the mean ± S.E. of six animals

^#^Significant at *p* < 0.05 compared to the control group; *significant at *p* < 0.05 compared to the CCl_4_-treated group

### Eliminated DPPH in hepatic tissue and serum of rats treated with *C. nudiflora*

As shown in Table 5, a significant reduction was noticed in the DPPH levels of the CCl_4_-treated group as compared to the normal control group. In contrast, a significant increase of DPPH levels was noticed in the hepatic tissue and serum of CCl_4_-administered rats treated with *C. nudiflora* at various doses.

**Table 5:** Eliminated DPPH in hepatic tissue and serum of rats treated with *C. nudiflora* at various doses

Groups	Eliminated tissue DPPH (%)	Eliminated serum DPPH (%)
Saline control	79.17 ± 1.41	34.42 ± 1.31
CCl_4_ (1 ml/kg b.wt.)	68.85 ± 1.29^#^	23.40 ± 1.86^#^
*C. nudiflora* (150 mg/kg b.wt.) + CCl_4_	73.89 ± 1.96*	26.26 ± 1.49*
*C. nudiflora* (300 mg/kg b.wt.) + CCl_4_	77.71 ± 1.21*	30.11 ± 0.84*
*C. nudiflora* (450 mg/kg b.wt.) + CCl_4_	77.77 ± 1.69*	31.20 ± 1.79*
*C. nudiflora* (450 mg/kg b.wt.)	78.96 ± 1.97*	34.03 ± 2.23*

Each value represents the mean ± S.E. of six animals

^#^Significant at *p* < 0.05 compared to the control group; *significant at *p* < 0.05 compared to the CCl_4_-treated group

### Eliminated ABTS in hepatic tissue and serum of rats with *C. nudiflora* treatment

Significant reduction was noticed in the ABTS levels of the CCl_4_-administered group as compared to the normal and plant control groups (Table 6). Meanwhile, a significant elevation of ABTS levels was recorded in the hepatic tissue and serum of CCl_4_-administered rats treated with *C. nudiflora*.

**Table 6:** Eliminated ABTS in hepatic tissue and serum of rats treated with *C. nudiflora* at various doses

Groups	Eliminated tissue ABTS (%)	Eliminated serum ABTS (%)
Saline control	82.07 ± 1.58	87.34 ± 1.06
CCl_4_ (1 ml/kg b.wt.)	63.86 ± 6.58^#^	79.22 ± 1.33^#^
*C. nudiflora* (150 mg/kg b.wt.) + CCl_4_	72.03 ± 6.31*	82.92 ± 0.80*
*C. nudiflora* (300 mg/kg b.wt.) + CCl_4_	75.79 ± 5.99*	84.03 ± 0.26*
*C. nudiflora* (450 mg/kg b.wt.) + CCl_4_	77.77 ± 3.13*	85.02 ± 1.07*
*C. nudiflora* (450 mg/kg b.wt.)	82.34 ± 1.32*	86.95 ± 2.80*

Each value represents the mean ± S.E. of six animals

^#^Significant at *p* < 0.05 compared to the control group; *significant at *p* < 0.05 compared to the CCl_4_-treated group

### Effect of *C. nudiflora* on CCl_4_-induced histopathological changes

#### Light microscopy observation

**Fig. 6:**
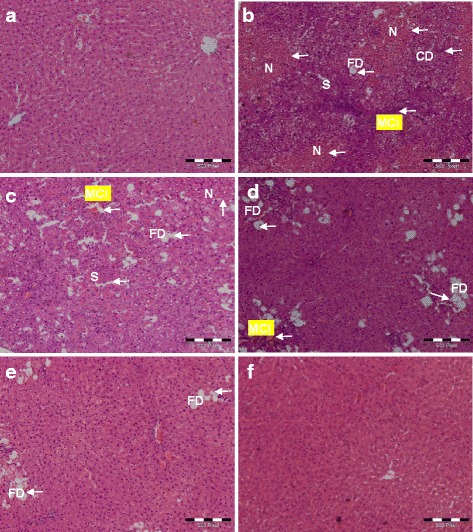
Effect of *C. nudiflora* on CCl4-induced histopathological changes (H&E staining). **a** Control group, normal histopathology. **b** CCl4 (1 ml/kg b.wt.) hepatic necrosis (N), mononuclear cell infiltration (MCI), fatty degeneration (FD), sinusoidal spaces (S), and cell derangement (CD). **c** *C. nudiflora* (150 mg/kg b.wt. + CCl_4_) repairing of hepatocytes. **d** *C. nudiflora* (300 mg/kg b.wt. + CCl_4_) repairing of hepatocytes. **e** *C. nudiflora* (450 mg/kg b.wt. + CCl_4_) repairing of hepatocytes. **f** *C. nudiflora* (450 mg/kg b.wt.) normal histopathology

Histological changes, including increased fatty degeneration, necrosis, blood vessel congestion, and derangement of hepatocytes, were observed in CCl_4_-treated rats alone. However, these changes were significantly improved by *C. nudiflora*, as shown in Fig. 6, which suggests that pretreatment with methanol extracts of *C. nudiflora* significantly prevented CCl_4_-induced hepatic injury.

#### Ultrastructural observations

**Fig. 7:**
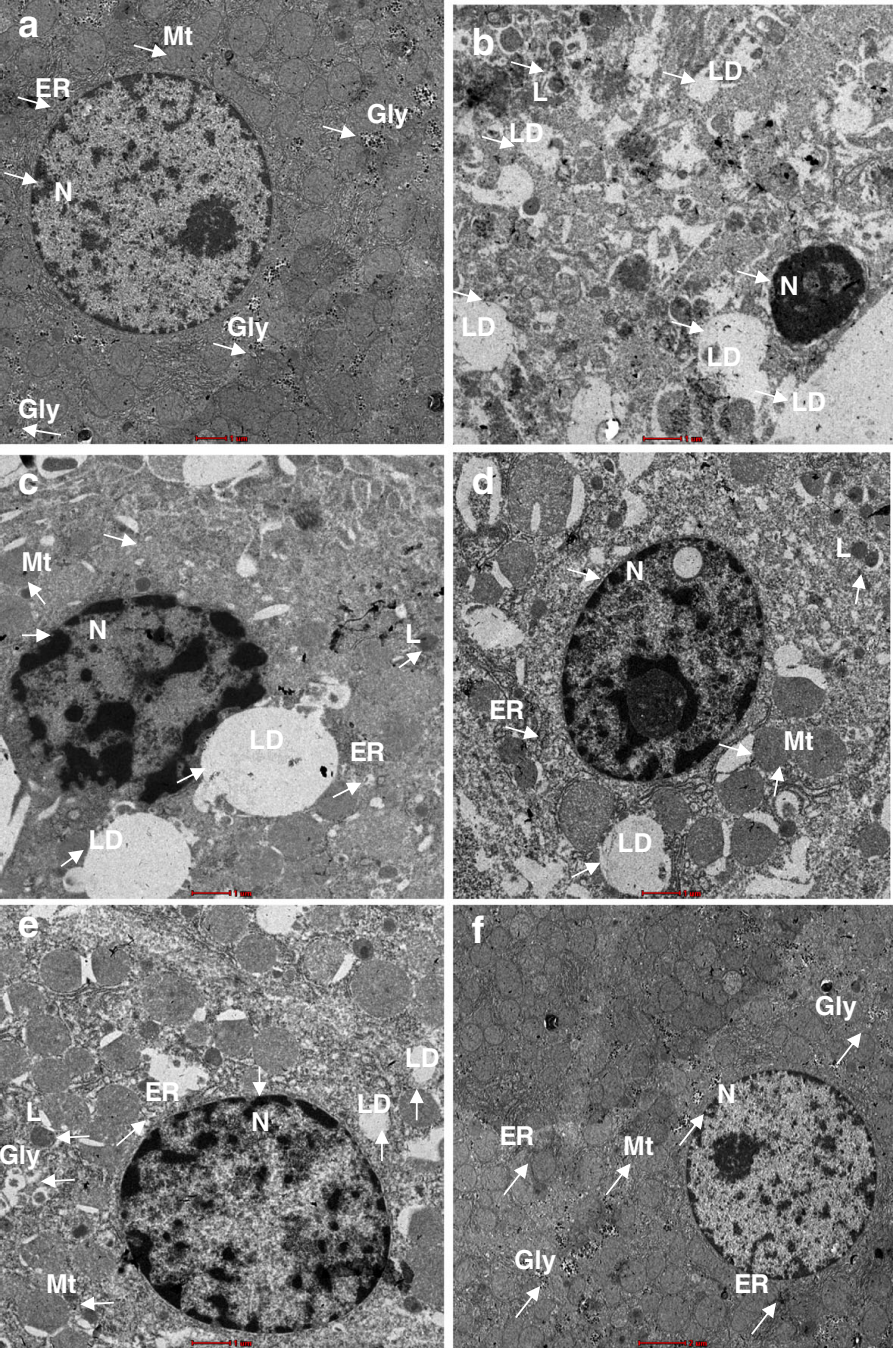
Protective effect of *C. nudiflora* on the ultrastructure of hepatocytes damaged by CCl4. **a** (×690) Control; hepatocytes show normal ultrastructure. **b** (×890) CCl4 (1 ml/kg b.wt.); glycogen loss, large lipid droplets (LD), degenerated nucleus, and disrupted endoplasmic reticulum are seen. **c** (×890) *C. nudiflora* (150 mg/kg b.wt. + CCl_4_); mitochondria are visible, and lipid droplets are present. **d** (×890) *C. nudiflora* (300 mg/kg b.wt. + CCl_4_); disrupted nuclear membrane and dilations in the endoplasmic reticulum are recovered, and lipid droplets are present. **e** (×89×) *C. nudiflora* (450 mg/kg b.wt. + CCl_4_); disrupted nuclear membrane and dilations in the endoplasmic reticulum are recovered, and lipid droplets are still present. The number of mitochondria and glycogen granules is increased. **f** (×550) *C. nudiflora* (450 mg/kg b.wt.) normal hepatocytes

The ultrastructure of liver sections of the normal control group showed regular nuclei with intact nuclear envelopes, endoplasmic reticulum at proximity to the nucleus, and abundant mitochondria in the cytoplasm (Fig. 7a). Ultrastructural analysis of the hepatocytes of the CCl_4_-intoxicated group demonstrated extensive cellular damage with irregular and damaged nuclei, significant dilations in ERs, large lipid globules, and glycogen loss (Fig. 7b). Pretreatment of the rats in group 3 with *C. nudiflora* significantly reduced the damage in hepatocytes. However, some lytic areas, lipid droplets, and dilations in ER were still noticed (Fig. 7c). Pretreatment of the rats in groups 4 and 5 with *C. nudiflora* provided overall improvement in the ultrastructure with intact nuclear envelopes, abundant mitochondria, and glycogen granules (Fig. 7d, e). The plant control group showed normal hepatocytes (Fig. 7f).

### Effect of *C. nudiflora* on CCl_4_-induced immunohistochemical changes

**Fig. 8:**
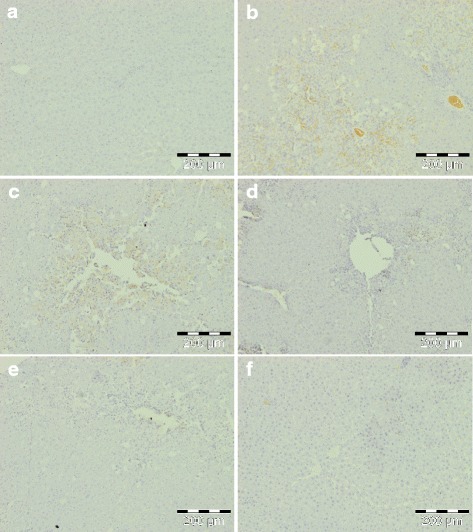
Immunohistochemical appearance of HNE-modified protein adducts. **a** Control group, normal. **b** CCl4 (1 ml/kg b.wt.), increased formation of HNE-modified protein adducts. **c** *C. nudiflora* (150 mg/kg b.wt. + CCl_4_), decreased formation of HNE-modified protein adducts. **d** *C. nudiflora* (300 mg/kg b.wt. + CCl_4_), decreased formation of HNE-modified protein adducts. **e** *C. nudiflora* (450 mg/kg b.wt. + CCl_4_), decreased formation of HNE-modified protein adducts. **f** *C. nudiflora* alone (450 mg/kg b.wt.), normal

Since HNE, a major aldehydic product of lipid peroxidation, is believed to be largely responsible for cytopathological effects observed during oxidative stress [1–3], we studied the effect of *C. nudiflora* against CCl_4_-induced formation of HNE-modified protein adducts in the liver. The production of HNE-modified protein adducts is indicated by the appearance of yellow coloration (Fig. 8). The CCl_4_-administered group showed intense yellow coloration as compared to the normal and plant control groups. However, very light coloration was noticed in the group treated by *C. nudiflora*.

**Fig. 9:**
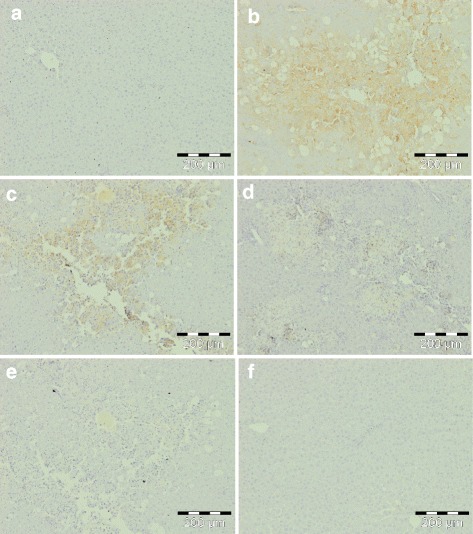
Immunohistochemical appearance of 8-OHdG. **a** Control group, normal. **b** CCl_4_ (1 ml/kg b.wt.), increased formation of 8-OHdG. **c** *C. nudiflora* (150 mg/kg b.wt. + CCl_4_), decreased formation of 8-OHdG. **d** *C. nudiflora* (300 mg/kg b.wt. + CCl_4_), decreased formation of 8-OHdG. **e** *C. nudiflora* (450 mg/kg b.wt. + CCl_4_), decreased formation of 8-OHdG. **f** *C. nudiflora* alone (450 mg/kg b.wt.), normal

8-OHdG, a DNA-based modified product, is one of the most commonly used markers for evaluation of oxidative DNA damage. Thus, we studied the effect of *C. nudiflora* against CCl_4_-induced formation of 8-OHdG in the liver. Figure 9 indicates the formation of 8-OHdG by the appearance of intense yellow in the CCl_4_-treated group as compared to the normal control group. This demonstrates that more 8-OHdG-positive cells are present. Meanwhile, the plant-treated group indicated faint yellow stains.

**Fig. 10:**
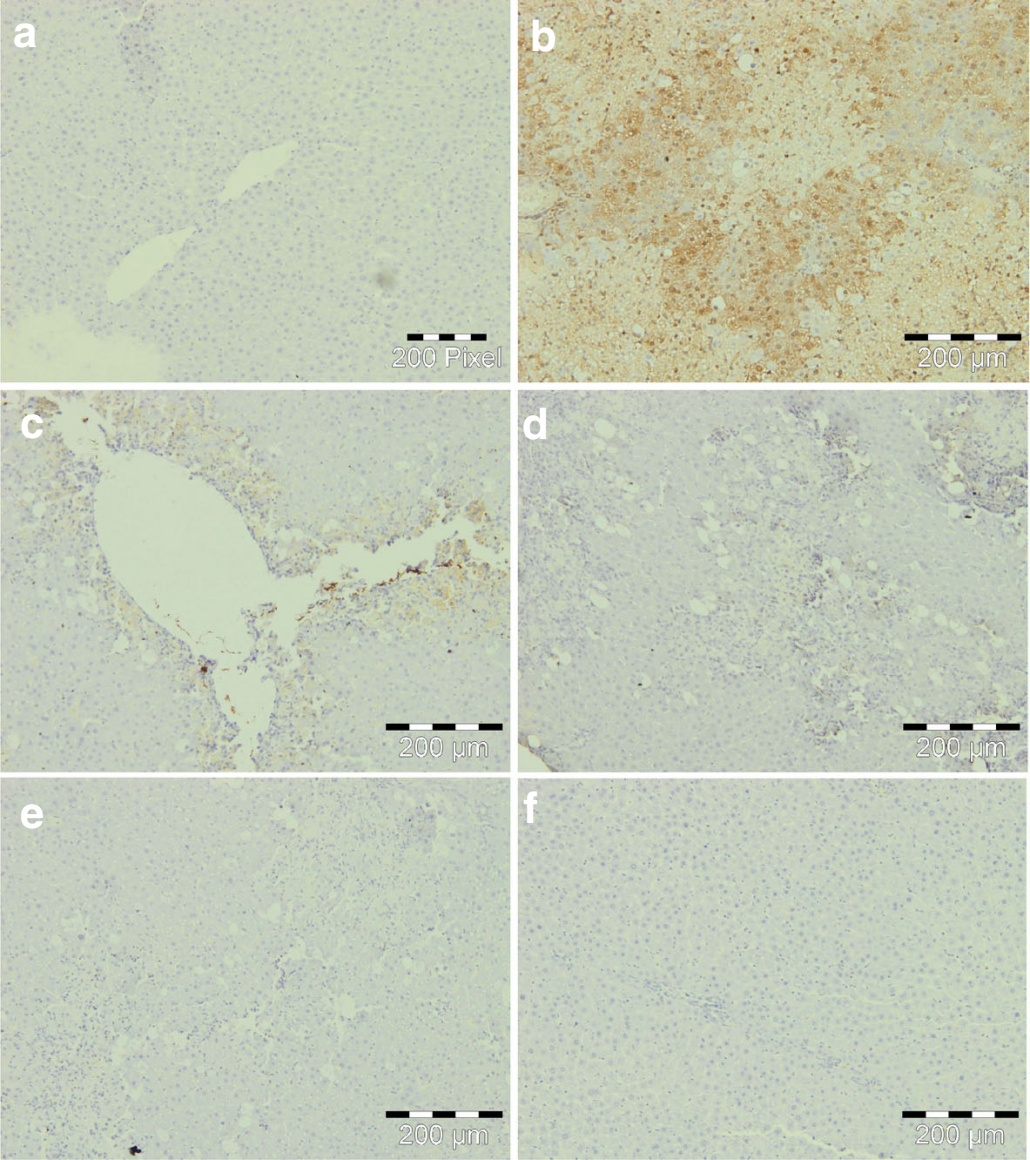
Immunohistochemical appearance of TNF-α. **a** Control group, normal. **b** CCl_4_ (1 ml/kg b.wt.), high expression of TNF-α. **c** *C. nudiflora* (150 mg/kg b.wt. + CCl_4_), low expression of TNF-α. **d** *C. nudiflora* (300 mg/kg b.wt. + CCl_4_), low expression of TNF-α. **e** *C. nudiflora* (450 mg/kg b.wt. + CCl_4_), low expression of TNF-α. **f** *C. nudiflora* alone (450 mg/kg b.wt.), normal

**Fig. 11:**
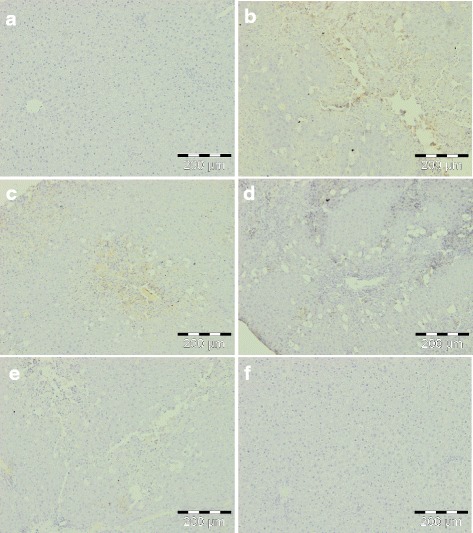
Immunohistochemical appearance of IL-6. **a** Control group, normal. **b** CCl_4_ (1 ml/kg b.wt.), high expression of IL-6. **c** *C. nudiflora* (150 mg/kg b.wt. + CCl_4_), low expression of IL-6. **d** *C. nudiflora* (300 mg/kg b.wt. + CCl_4_), low expression of IL-6. **e** *C. nudiflora* (450 mg/kg b.wt. + CCl_4_), low expression of IL-6. **f** *C. nudiflora* alone (450 mg/kg b.wt.), normal

**Fig. 12:**
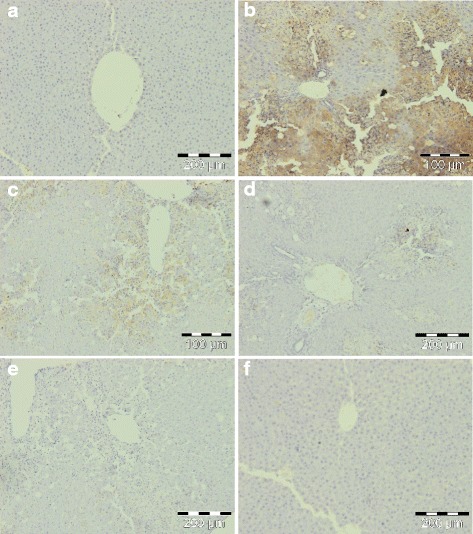
Immunohistochemical appearance of PGE2. **a** Control group, normal. **b** CCl_4_ (1 ml/kg b.wt.), high expression of PGE2. **c** *C. nudiflora* (150 mg/kg b.wt. + CCl_4_), low expression of PGE2. **d** *C. nudiflora* (300 mg/kg b.wt. + CCl_4_), low expression of PGE2. **e** *C. nudiflora* (450 mg/kg b.wt. + CCl_4_), low expression of PGE2. **f** *C. nudiflora* alone (450 mg/kg b.wt.), normal

CCl_4_-induced hepatotoxicity is accompanied by the excessive production of proinflammatory mediators such as TNF-α, IL-6, and PGE2. Therefore, the effect of *C. nudiflora* on the expression of proinflammatory markers like TNF-α, IL-6, and PGE2 was also analyzed (Figs. 10, 11, and 12). The liver sections of the normal and plant control groups showed complete absence of immunostaining of TNF-α, IL-6, and PGE2. A large amount of TNF-α-, IL-6-, and PGE2-immunopositive cells was noticed in the hepatic tissue sections of CCl_4_-administered rats as compared to control rats. In contrast, a decrement of TNF-α, IL-6, and PGE2 immunopositivity was observed in *C. nudiflora*-treated rats.

## Discussion

In this study, a GCMS analysis of the bioactive compounds in *C. nudiflora* extracts revealed the presence of bioactive compounds with antioxidant, anti-inflammatory, anticancer, antimicrobial, and hepatoprotective properties. It is widely recognized that dietary antioxidants have positive influences in reducing the risk of development of nutrition-related chronic diseases [25], and this fact has stimulated the search for new antioxidant sources. In the present study, the hepatoprotective effects of *C. nudiflora* were first investigated by suppressing CCl_4_-induced oxidative stress and inflammation in the livers of rats and attenuating the morphological changes caused by CCl_4_. Our findings contribute to the understanding that *C. nudiflora* treatment reduces the occurrence of liver oxidative injuries, and this nutritional strategy presents an alternative to current pharmaceutical approaches aimed at reducing hepatotoxicity.

Herein, nine bioactive compounds have been identified from methanol extracts of *C. nudiflora*. Among the identified compounds is phenol, a simple phenolic compound. It has been reported in the ethanol bark extract of *Ficus religiosa* Linn with antioxidant, antiseptic, and antibacterial properties [26]. Benzyl alcohol, an aromatic compound, has been also noticed in extracts of basil leaves (*Ocimum basilicum* L.) and reported with antioxidant and antimicrobial activities [27–29]. Eugenol, a phenolic compound, has been also detected in extracts of basil leaves (*O. basilicum* L.) and *Eugenia caryophyllata*. The antioxidant and anti-inflammatory activities of the compound have been reported [27–31]. Phenol,2,4-bis(1,1-dimethylethyl), an alkylated phenol, has been detected in extracts of *Plumbago zeylanica*, *Hybanthus enneaspermus*, and *Tephrosia tinctoria* and reported with antioxidant, anticancer, and antimicrobial properties [32, 33]. The abovementioned bioactive compounds have been reported with antioxidant and anti-inflammatory properties. Thus, we believe that these compounds have played a vital role in the protection of liver by neutralizing the free radicals produced by the administration of CCl_4_.

Dodecanoic acid, a lauric and saturated fatty acid, has been found in extracts of *Vitex altissima* L. The compound has been reported with antimicrobial, antioxidant, antiviral, and hypocholesterolemic properties [34–36]. Hexadecanoic acid (ethyl ester), a palmitic acid ester, has been detected in *Vitex negundo* and reported with antioxidant and hypocholesterolemic properties [37]. *N*-Hexadecanoic acid, a palmitic acid (saturated fatty acid), has been noticed in *Centaurea aladagensis* and reported with antioxidant, hypocholesterolemic, and hemolytic activities [37, 38]. Phytol, a diterpene alcohol, has been also found in the *H. enneaspermus* and reported with antimicrobial, anticancer, anti-inflammatory, hepatoprotective, and diuretic activities [37–39]. 9,12-Octadecadienoic acid, commonly known as linoleic acid (unsaturated fatty acid), has been also detected in *Scotia brachypetala* and reported with anti-inflammatory, anticancer, hypocholesterolemic, and hepatoprotective properties [37, 40, 41].

The study also demonstrates the hepatoprotective and antioxidative effects of *C. nudiflora* against CCl_4_-induced oxidative hepatic damage in rats. CCl_4_ is a known hepatotoxin, mostly utilized for the induction of hepatic injuries in experimental animals [42]. In our study, it is indicated that CCl_4_ results in a moderate decrease in body weight as compared to the control group. During the experiment, a constant increase in the body weight in the control group was noticed for 15 days with no mortality in all groups. However, sudden changes were noticed in the food and water intake of the CCl_4_-administered group alone after CCl_4_ treatment on the 13th and 14th days, which resulted in a decrease in body weight. The changes were less obvious in rats which were pretreated with *C. nudiflora* as compared to the CCl_4_-treated group. However, no changes were noticed in the food and water intake of the plant-treated control group (Table 2). Our results are in agreement with previous studies [43]. Thus, it is indicated that the methanol extract of *C. nudiflora* is effective in reducing the toxicity induced by CCl_4_.

CCl_4_ elevated levels of ALT and AST in the serum of the CCl_4_-treated rats, showing hepatic injury as these enzymes leak out from the liver into the blood due to hepatic tissue damage, which is always associated with hepatonecrosis [44, 45]. With the administration of *C. nudiflora* at various doses, the levels of these marker enzymes were restored in a dose-dependent fashion. The recovery of these damages may be due to the stabilization of the plasma membrane and the repair of hepatocytes. The results indicate that treatment with methanol extracts of *C. nudiflora* could protect the liver against damage caused by CCl_4_.

Lipid peroxidation is the main mechanism of hepatic damage. CCl_4_ is biotransformed by the catalytic activity of the liver cytochrome P450 in the endoplasmic reticulum to generate free radicals, mainly trichloromethyl (CCl_3_). Free radicals react with oxygen to produce trichloromethyl peroxyl (CCl_3_O_2_·) radicals. The resulted toxic metabolites have the potential to bind to various proteins or lipids and initiate the peroxidation of lipids [46, 47]. In the current study, both trichloromethyl and peroxyl radicals seem to initiate the degradation of membrane lipids. This causes the generation of lipid peroxides, which, in turn, give MDA products that result in a loss of cell membrane integrity and liver injury [46, 47]. Pretreatment of *C. nudiflora* methanol extracts markedly reduced the generation of the lipid peroxidation end product (MDA) in a dose-dependent manner. This shows that the administration of *C. nudiflora* methanol extracts effectively minimized lipid peroxidation induced by CCl_4_.

GSH, a well-known antioxidant, plays an important role against CCl_4_-induced injury by covalently binding to CCl_3_· radicals and enhancing the activities of glutathione peroxidase and glutathione reductase [46, 48]. Pretreatment with *C. nudiflora* methanol extracts resulted in elevating the liver GSH levels by 23, 34, and 38% as compared with the CCl_4_-treated group. The GSH levels in animals pretreated with *C. nudiflora* show no significant differences when compared with the saline-treated control group.

CCl_4_ intoxication also affects the activities of hepatic antioxidant enzymes. All oxygen-utilizing organisms are equipped with well-organized antioxidant systems to prevent damage caused by free radicals. These enzymes include catalase, glutathione peroxidase, glutathione reductase, glutathione-6-phosphate dehydrogenase, glutathione *S*-transferase, and quinone reductase. These enzymes act as the first line of defense to counteract free radical-induced oxidative stress [49]. Glutathione peroxidase is an enzyme which minimizes the production of peroxide radicals (hydrogen peroxide and alkyl hydroperoxides) in association with GSH [50]. Catalase neutralizes harmful H_2_O_2_ to oxygen and water. Glutathione *S*-transferase and quinone reductase (phase II detoxification enzymes) increase cellular GSH levels and protect cells against the toxicities of electrophiles [51]. Glutathione-6-phosphate dehydrogenase is involved in the generation of NADPH through pentose phosphate pathways. NADPH is required for the production of GSH, which is necessary for cell protection against free radical damage [52]. The pretreatment of rats with various doses of *C. nudiflora* methanol extracts significantly increased the activities of these antioxidant enzymes, as compared with the CCl_4_ alone-treated group. Using DPPH and ABTS assays, we found that methanol extracts of *C. nudiflora* increased DPPH and ABTS levels in the liver and serum of CCl_4_-treated rats. Our findings are in agreement with previously published data [53].

Light microscopy analysis demonstrated that animals treated with CCl_4_ alone showed marked destruction of liver architecture with extensive fatty degeneration, blood vessel congestion, derangement of the hepatic cells, and necrosis, while the saline- and plant-treated control groups showed normal hepatic cells with intact cytoplasm, prominent nuclei, and visible central veins. However, the pretreatment of animals with various doses of *C. nudiflora* reduced the histopathological changes and resulted in less-pronounced destruction of liver architecture, which indicates that pretreatment with methanol extracts of *C. nudiflora* reduced liver injuries.

Ultrastructural findings that were pointed out in the CCl_4_-treated group include dilations and irregular organization of membranes, large lipid droplets, and glycogen loss. The changes in organelle structure and edematous cytoplasmic matrix were probably due to alterations in membrane structure caused by lipid peroxidation. Our results are in agreement with previous reports [54]. MDA levels of the CCl_4_-treated group were more elevated when compared to the normal control group, which further supports these histological results. Administration of *C. nudiflora* markedly reduced the cellular damage in CCl_4_-treated rats.

The administration of CCl_4_ also markedly increased the formation of HNE-modified protein adducts and 8-OHdG. HNE is a major aldehyde product of lipid peroxidation and displays a variety of cytopathological properties, including the inhibition of enzymes and proteins, as well as DNA and RNA synthesis [55]. The aldehyde is highly toxic to hepatocytes. In addition to this, it also has genotoxic and mutagenic effects. It is believed that the toxicity of HNE is due to its reactive aldehyde group. On the other hand, 8-OHdG is a major product of oxidative DNA damage. Toyokuni et al. [56] and Uchida et al. [57] have reported that the process of lipid peroxidation has been involved in promoting the development of 8-OHdG by the production of HNE-modified protein adducts. Thus, we were interested to investigate whether various doses of *C. nudiflora* were able to block the increase in formation of HNE-modified protein adducts and 8-OHdG in the livers of CCl_4_-treated rats. In the current research, the productions of HNE-modified protein adducts and 8-OHdG are indicated by yellow and brown coloration due to staining. Normal and plant-treated control groups reveal no yellow coloration which signifies that protein adducts and 8-OHdG are not produced, while the CCl_4_-intoxicated group displays intense yellow coloration as compared to normal and plant-treated control groups, which proves that CCl_4_ intoxication triggers the production of four HNE-modified protein adducts and 8-OHdG. Meanwhile, the administration of *C. nudiflora* methanolic extracts reduced the yellow coloration in CCl_4_-intoxicated groups in a dose-dependent fashion as compared to the CCl_4_ only-treated group (Figs. 8 and 9). Our results indicated that prophylactic treatments of *C. nudiflora* to rats can efficiently attenuate this increase. This shows that phytochemical compounds may be responsible for the biological effects of *C. nudiflora*.

In the current study, we also investigated the inhibitory effects of *C. nudiflora* on the expression of proinflammatory markers such as TNF-α, IL-6 (cytokines), and PGE2. Cytokines act as central regulators, controlling genes, and are accountable for causing either apoptosis or protective action on cells by stimulating the proliferation of hepatocytes. They also play an important role in the constitution of a complex network involved in the regulation of inflammatory responses. Important hepatotoxic mediators in various experimental models of hepatic damage are TNF-α, IL-6, and PGE2. These markers are formed and released from several cells under physiological and pathological stress, and the liver is highly vulnerable to the action of these markers [58]. These markers have been selected due to an important role in inflammation, vascular permeability, as well as proliferation [59–61]. In our findings, the markers were expressed in inflammatory cells. The overexpression of proinflammatory markers is indicated by intense yellow and brown coloration due to staining (Figs. 10, 11, and 12). The hepatic sections of the normal and plant control groups showed a complete absence of immunostaining of TNF-α, IL-6, and PGE2 which implies that no proinflammatory markers were expressed. On the other hand, a large amount of immunopositive cells was noticed in the hepatic tissue sections of the CCl_4_-administered rats as compared to the control rats. This demonstrates that more proinflammatory markers were expressed in the CCl_4_-treated group. The overexpression of TNF-α, IL-6, and PGE2 in the CCl_4_-administered rats was markedly reduced by *C. nudiflora* in a dose-dependent manner. Thus, this demonstrates that administration of *C. nudiflora* had anti-inflammatory effects via suppression of proinflammatory mediator expressions.

## Conclusion

Our data demonstrates that methanol extracts of *C. nudiflora* are good sources of bioactive compounds with antioxidant, anti-inflammatory, antimicrobial, antitumor, and hepatoprotective properties. In addition to this, extracts of *C. nudiflora* indicate effective protection against CCl_4_-induced liver injury in rats and resulted in the restoration and reduction of GSH and MDA contents in liver cells. It improved serum ALT and AST levels (hepatic enzyme markers) and increased antioxidant enzyme activities. A histopathology of the livers indicates that *C. nudiflora* reduced the incidence of hepatic lesions induced by CCl_4_. Furthermore, immunohistochemical studies indicate that the plant extracts decrease the formation of HNE-modified protein adducts and 8-OHdG and also reduce the expression of TNF-α, IL-6, and PGE2 in liver cells. These results indicate that *C. nudiflora* extracts play a protective role in CCl_4_-induced liver injury, which might be due to elevated antioxidant defense potentials, suppressed inflammatory response, and oxidative stress of liver tissues. These findings exhibit the potential prospects of *C. nudiflora* as a functional ingredient to prevent ROS-related liver damage.

## Abbreviations

8-OHdG: 8-Hydroxy-2′-deoxyguanosine
ALT: Alanine transaminase
AST: Aspartate transaminase
GSH: Glutathione
HNE: 4-Hydroxy-2-nonenal
IL-6: Interleukin 6
MDA: Malondialdehyde
PGE2: Prostaglandin E2
TNF-α: Tumor necrosis factor alpha
